# Association between fatty liver and risk of liver failure in patients with acute hepatitis B: a retrospective cohort study

**DOI:** 10.3389/fcimb.2026.1712115

**Published:** 2026-01-27

**Authors:** Xiao-hao Wang, Yu-nan Chang, Lu Zhang, Yan-li Yang, Lu-wen Liang, Yi Zeng, Zhi Zhou, Shan Zhong, Hu Li

**Affiliations:** 1 Department of Infectious Diseases, Key Laboratory of Molecular Biology for Infectious Diseases, Ministry of Education, Institute for Viral Hepatitis, The Second Affiliated Hospital, Chongqing Medical University, Chongqing, China; 2 Department of Infectious Diseases, Children’s Hospital of Chongqing Medical University, Chongqing, China; 3 National Clinical Research Center for Children and Adolescents' Health and Diseases, Ministry of Education Key Laboratory of Child Development and Disorders, Chongqing Key Laboratory of Child Rare Diseases in Infection and Immunity, Chongqing, China

**Keywords:** acute hepatitis B, fatty liver, HBsAg loss, liver failure, seroconversion

## Abstract

**Background and aims:**

Fatty liver (FL) is a common comorbidity that has been associated with adverse clinical outcomes in various liver diseases. However, its impact on the prognosis of acute hepatitis B (AHB) remains unclear. This study aimed to evaluate the influence of FL on liver-related outcomes among hospitalized patients with AHB.

**Methods:**

A retrospective analysis was conducted on hospitalized patients diagnosed with AHB from January 1, 2010, to December 30, 2023. Demographic and clinical data were collected, and patients were categorized into AHB with FL (AHB-FL) and AHB without FL (AHB-no FL) groups based on imaging and laboratory examinations. Multivariate regression models were utilized to investigate the association between FL and liver-related outcomes, including acute liver failure (ALF), hepatitis B surface antigen (HBsAg) loss, and seroconversion. Kaplan-Meier analysis was performed to assess differences in time to HBsAg loss and seroconversion.

**Results:**

A total of 200 eligible patients were included, with 29 (14.5%) in the AHB-FL group and 171 (85.5%) in the AHB-no FL group. The incidence of ALF was significantly higher in the AHB-FL group (34.5% vs. 15.8%, P = 0.02). No significant differences were observed in rates of HBsAg loss (75.9% vs. 82.5%, P = 0.40) or seroconversion (44.4% vs. 53.1%, P = 0.40) between the two groups. Kaplan-Meier analysis indicated comparable times to HBsAg loss (P = 0.07) and seroconversion (P = 0.43). Multivariable analysis confirmed FL as an independent risk factor for ALF (OR 4.61, 95% CI 1.26–16.86; P = 0.02), but not for HBsAg loss (HR 1.60, 95% CI 0.92–2.78; P = 0.10) or seroconversion (HR 1.69, 95% CI 0.80–3.57; P = 0.17). Subgroup analysis indicated a stronger association between FL and ALF in rural patients (P interaction=0.02), suggesting regional differences may affect clinical risks and management.

**Conclusion:**

FL constitutes a robust and independent determinant of ALF in AHB patients, sustained after rigorous adjustment for confounding factors. This evidence highlights a specific metabolic aggravation of liver injury, mandating the integration of FL screening into early risk stratification and management protocols.

## Graphical Abstract

**Figure:**
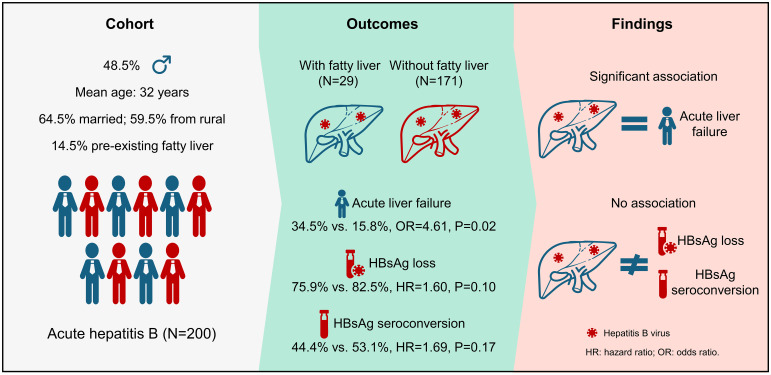

## 1 Introduction

Hepatitis B virus (HBV) infection remains a significant global health challenge, causing approximately 1.2 million new cases and an estimated 1.1 million deaths annually, primarily due to complications such as cirrhosis and acute liver failure (ALF) (World Health Organization, 2024). While most (>95%) adults infected with HBV recover spontaneously and do not require specific treatment, about 0.5% progress to ALF (Burns and Thompson, 2014; Maiwall et al., 2024). The pathogenesis of HBV-induced ALF involves a complex interplay of viral factors and dysregulated host immunity, including basal core promoter or pre-core variants, excessive B-cell activation, and tumor necrosis factor-alpha/interferon-gamma mediated hepatocyte apoptosis (Burns and Thompson, 2014; Guidotti et al., 2015; Iannacone and Guidotti, 2022; Vanwolleghem et al., 2022). Although some risk factors have been identified, the significant burden of ALF underscores the need to identify additional risk factors to provide a comprehensive understanding of acute hepatitis B (AHB) management (Patterson et al., 2020; Liu et al., 2022; Su et al., 2022).

The global rise in obesity has significantly increased the prevalence of fatty liver (FL) across populations and age groups, with nearly 40% of adults affected (Hirode and Wong, 2020; Chan et al., 2022; Paik et al., 2022; Huang et al., 2025). Emerging evidence indicates that FL is not only a key modifier of the severity of liver injury but is also increasingly recognized as a risk factor for various adverse clinical outcomes (Yip et al., 2022; Choudhury et al., 2024; Jiang et al., 2024; Huang et al., 2025; Zhong et al., 2025). In populations with chronic HBV infection, observational studies have found inconsistent associations between coexisting FL and liver-related outcomes (Mak et al., 2020; Li et al., 2021; Mak et al., 2021; Kim et al., 2022; Yang and Wei, 2022; Huang and Liu, 2023; Huang et al., 2023). A recent meta-analysis demonstrated that among chronic hepatitis B (CHB) patients, the presence of FL (vs. no FL) was associated with significantly lower risks of cirrhosis, hepatocellular carcinoma, and mortality, alongside a higher probability of hepatitis B surface antigen (HBsAg) seroconversion (Wong et al., 2023). Ongoing discussions address the diverse effects of FL on the natural history of CHB infection; however, the impact of FL on outcomes in AHB remains virtually unexplored. Therefore, exploring the role of FL in AHB becomes increasingly important.

Although it is well known that acute HBV infection increases the risk of adverse liver outcomes, data regarding how concomitant FL modifies this prognosis remains scant. To address this knowledge gap, we designed a retrospective cohort study specifically structured to isolate the independent contribution of FL to AHB outcomes. Our analytical trajectory moves beyond simple correlation; we utilized stepwise multivariate regression models to rigorously control for demographic and clinical confounders, thereby testing the central hypothesis that FL acts as a distinct driver of hepatocellular failure. By systematically contrasting ALF risk against viral clearance outcomes, this study aims to disentangle the metabolic impact on liver injury from viral immunological responses, providing a solid evidence base for precise risk stratification.

## 2 Materials and methods

### 2.1 Study design and patient cohort

This was a retrospective cohort study of consecutive hospitalized patients with AHB at the Infectious and Liver Disease Center of the Second Affiliated Hospital of Chongqing Medical University, China, from January 1, 2010, to December 30, 2023. Patients with AHB were assessed at baseline and then followed up every 2 to 4 weeks for 24 weeks, with the schedule adjusted based on the resolution of the illness and extended if possible. The criteria for diagnosis of AHB included: (1) clinical picture consistent with AHB; (2) positive detection of serum IgM anti-HBc; (3) positive HBsAg test with clear evidence showing that serum HBsAg was negative within the past 6 months; and (4) absence of evidence of pre-existing infection and HBV reactivation. The study excluded patients based on the following criteria: (1) co-infection with hepatitis A virus (HAV), hepatitis C virus (HCV), hepatitis E virus (HEV), human immunodeficiency virus (HIV), or other similar infections; (2) presence of cirrhosis, liver cancer, or other malignancies; (3) pregnancy; and (4) incomplete follow-up data.

This study was approved by the Ethics Committee of the Second Affiliated Hospital of Chongqing Medical University and conducted in accordance with the ethical guidelines of the Declaration of Helsinki (No.202557). The data was anonymized and the requirement for informed consent was waived.

### 2.2 Measures of variables

Medical history, physical examination, health surveys, and blood tests were part of baseline evaluation for each patient. Demographic data collected included age, sex, smoking status (categorized as current smoker, or non-smoker), alcohol consumption (defined as regular intake of alcohol exceeding the recommended limits), marital status (single, married, or divorced), and residential area (urban or rural). Laboratory data were obtained from the central laboratory records and included complete blood count, liver function tests, international normalized ratio (INR), alpha fetoprotein, HBV DNA level, HBV markers, as well as serological tests for antibodies to HAV, HCV, HEV, and HIV. Serum alanine aminotransferase (ALT) levels were measured using sex-specific upper limits of normal (ULN), set at 40 U/L for females and 50 U/L for males, while the ULN for serum aspartate aminotransferase (AST) is 40 U/L. HBV DNA levels were determined using quantitative polymerase chain reaction, with a lower limit of detection of 20 IU/ml. The HBV markers analyzed included HBsAg (reference value < 0.05 IU/ml), anti-HBs (reference value < 10 mIU/ml), HBeAg, anti-HBe, and anti-HBc.

### 2.3 Diagnosis of fatty liver and ALF

The diagnosis of fatty liver (FL) was adjudicated by two imaging specialists based on imaging examinations (ultrasonography, computed tomography, or magnetic resonance imaging) using standardized diagnostic criteria (Leung et al., 2023). Specifically, FL was defined by typical imaging features of hepatic steatosis on the corresponding modality (e.g., increased hepatic echogenicity and hepatorenal contrast on ultrasonography; reduced liver attenuation and/or liver–spleen attenuation difference on computed tomography; or signal changes consistent with fat deposition on magnetic resonance imaging), as described in the referenced diagnostic guidance (Leung et al., 2023).

ALF was diagnosed based on the Asian Pacific Association for the Study of the Liver (APASL) guidelines, which include the rapid onset of severe liver dysfunction, hepatic encephalopathy, jaundice (serum total bilirubin ≥ 171 μmol/L or a daily increase ≥ 17.1 μmol/L), and coagulopathy (prothrombin activity ≤ 40% or INR ≥ 1.5) (Sarin et al., 2019).

### 2.4 Study outcomes

The primary outcome was the occurrence of ALF events in patients with AHB. Secondary outcomes included HBsAg loss (defined as the transition from HBsAg positive to negative) and seroconversion (defined as the appearance of anti-HBs).

### 2.5 Statistical analysis

Continuous variables are reported as means with standard deviations or medians with interquartile ranges, depending on their distribution. Differences in continuous variables between groups with and without FL (AHB-FL vs. AHB-no FL) were assessed using the Student t-test or the Mann-Whitney U test. Categorical variables (sex, smoking status, alcohol consumption, marital status, residential area, and HBeAg status) are expressed as frequencies and percentages, with differences analyzed using the chi-square test.

Kaplan-Meier curves were used to estimate the cumulative proportions of HBsAg loss and seroconversion. The time to HBsAg loss was defined as the interval between the adjudicated date of the acute event and the first date of HBsAg negativity. Similarly, time to HBsAg seroconversion was defined as the interval between the adjudicated date of the acute event and the first date of anti-HBs positivity. Group comparisons were performed using the log-rank test.

For the primary objective, the associations between FL (binary exposure variable: with or without) and ALF events (binary outcome variable) were assessed using binary logistic regression models. For the secondary objective, the associations between FL and HBsAg loss or seroconversion (binary outcome variables) were assessed using Cox proportional hazards regression models. Three models were applied: model 1 was unadjusted; model 2 was adjusted for age (as a continuous variable), sex, smoking status, and alcohol consumption (both as binary variables); and model 3 included further adjustments for laboratory and clinical variables, including ALT, AST, HBV DNA level, albumin, and the symptom onset to first medical contact (SO-to-FMC) (all as continuous variables). Data are presented as point estimates and corresponding 95% confidence intervals (CIs) of the effect size estimates.

Additionally, stratified analysis were performed in pre-specified subgroups defined by sex (male vs. female), smoking status (current smoker vs. non-smoker), alcohol consumption (drinker vs. non-drinker), marital status (single vs. other), residential area (urban vs. rural), HBV DNA level (<5 log_10_ IU/mL vs. >5 log_10_ IU/mL), HBeAg status (positive vs. negative), and SO-to-FMC (<5 days vs. >5 days). For each subgroup, the odds ratio (OR) and 95% CI for ALF in patients with AHB-FL (vs. AHB-no FL) were calculated. The p-value for interaction was also determined to assess whether the association between FL and ALF varied significantly across subgroups.

All analysis were performed with the use of R (http://www.R-project.org, R Foundation) and EmpowerStats (http://www.empowerstats.com, X&Y Solutions, Inc., Boston, MA, USA). A P value of less than 0.05 (two-sided) was considered to indicate statistical significance.

## 3 Results

### 3.1 Characteristics of the patients

**Figure 1:**
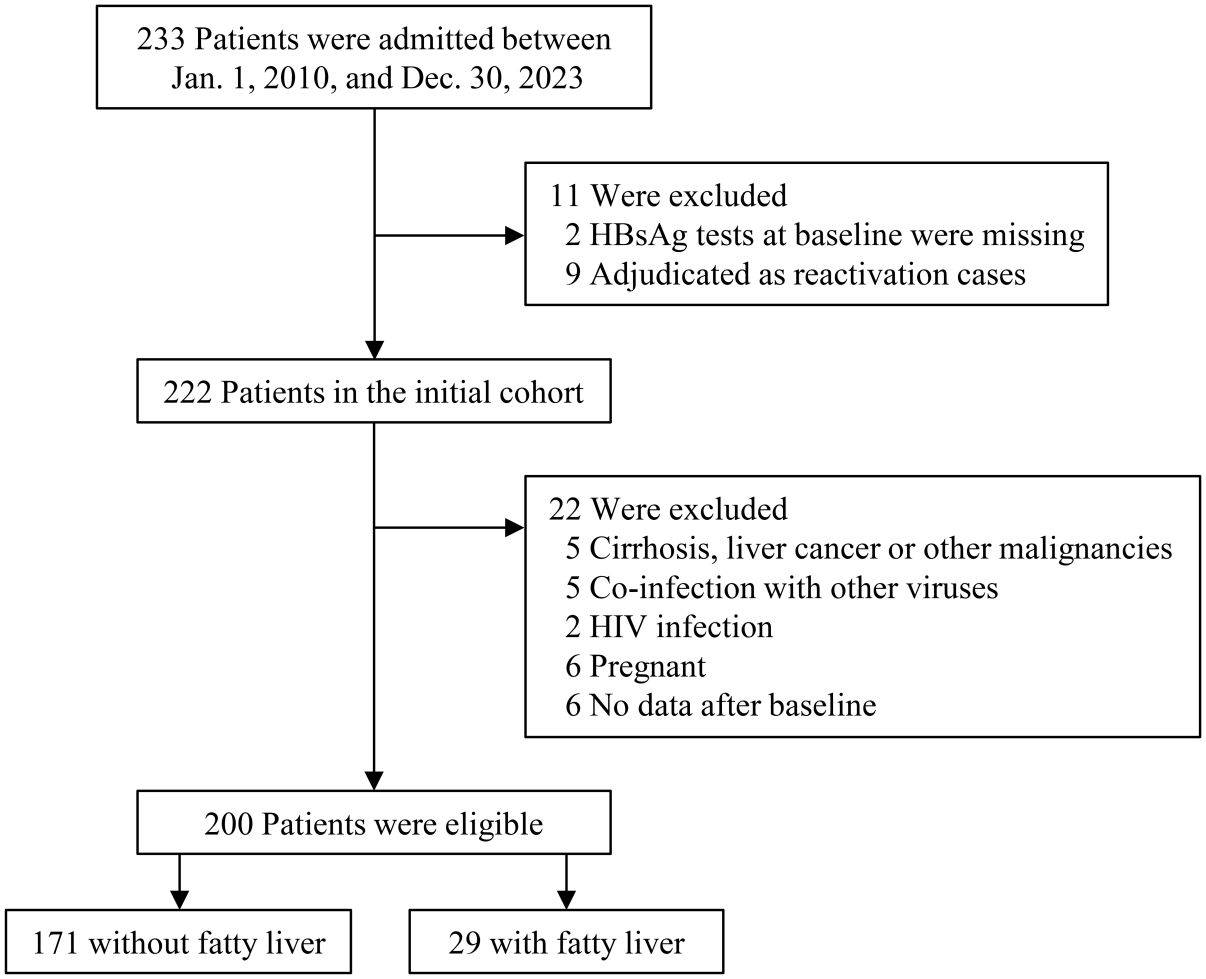
Flowchart for inclusion in the analytic cohort of patients. The numbers represent the number of cases. Other viruses include HAV, HCV, HEV, and Epstein-Barr virus. Abbreviations: HAV, hepatitis A virus; HCV, hepatitis C virus; HEV, hepatitis E virus; HBsAg, hepatitis B surface antigen; HIV, human immunodeficiency virus.

Between January 2010 and December 2023, a total of 233 patients with AHB were admitted. Based on the inclusion and exclusion criteria, 200 eligible patients were included in the final analysis, comprising 171 patients with AHB-no FL and 29 patients with (**Figure 1**). **Table 1** outlines the characteristics of these two groups. AHB-FL patients (vs. AHB-no FL) were significantly older (36 years vs. 29 years, p=0.02) and had a higher prevalence of males (79.3% [23/29] vs. 43.3% [74/171], p<0.001). A greater proportion of AHB-FL patients reported alcohol consumption (69.0% [20/29] vs. 29.8% [51/171], p<0.001). Additionally, AHB-FL patients exhibited higher levels of ALT, AST, and bilirubin compared to AHB-no FL patients (**Table 1**).

**Table 1:** Comparison of characteristics between the patients with and without fatty liver in this study.

Variables	Patients without FL (n=171)	Patients with FL (n=29)	P-value
Age, years	29.0 (24.0-36.5)	36.0 (27.0-48.0)	0.02
Male sex, n (%)	74 (43.3)	23 (79.3)	<0.001
Current smoker, n (%)	36 (21.1)	10 (34.5)	0.11
Alcohol drinking, n (%)	51 (29.8)	20 (69.0)	<0.001
Marital status, n (%)			0.76
Single	60 (35.1)	9 (31.0)	
Married	109 (63.7)	20 (69.0)	
Divorced	2 (1.2)	0 (0)	
Residential area, n (%) ^*^			0.92
Urban	69 (40.4)	12 (41.4)	
Rural	102 (59.6)	17 (58.6)	
Laboratory tests			
ALT, U/L	1511.0 (834.5-2303.5)	1852.0 (1670.0-3038.0)	0.01
ALT×ULN	34.1 (18.9-50.4)	37.0 (33.5-66.0)	0.04
AST, U/L	772.0 (289.5-1209.5)	912.0 (644.0-1573.0)	0.07
AST×ULN	19.3 (7.2-30.2)	22.8 (16.1-39.3)	0.07
Total bilirubin, µmol/L	88.9 (44.5-135.5)	117.3 (90.1-171.7)	0.01
Total protein, g/L	70.0 ± 7.7	71.9 ± 8.1	0.27
Albumin, g/L	40.2 ± 5.2	39.2 ± 4.6	0.34
INR	1.1 (1.0-1.3)	1.1 (1.0-1.8)	0.07
HBV DNA, log_10_ IU/mL	3.7 (2.7-4.8)	3.5 (2.7-4.8)	0.51
HBeAg, n (%)			0.45
Negative	64 (37.4)	13 (44.8)	
Positive	107 (62.6)	16 (55.2)	
SO-to-FMC, days	5.5 (3.2-8.0)	5.0 (2.8-7.0)	0.28

Data are presented as mean (standard deviation), median (interquartile range), or n (%).

^*^The urban-rural classification of the county of residence is based on the National Bureau of Statistics' Urban-Rural Classification Scheme for Counties. Abbreviations: ALT, alanine aminotransferase; AST, aspartate aminotransferase; DNA, deoxyribonucleic acid; FL, fatty liver; HBeAg, hepatitis B e antigen; HBsAg, hepatitis B surface antigen; HBV, hepatitis B virus; INR, international normalized ratio; SO-to-FMC, symptom onset to first medical contact time; ULN, upper limit of normal.

### 3.2 Primary and secondary outcomes

**Figure 2:**
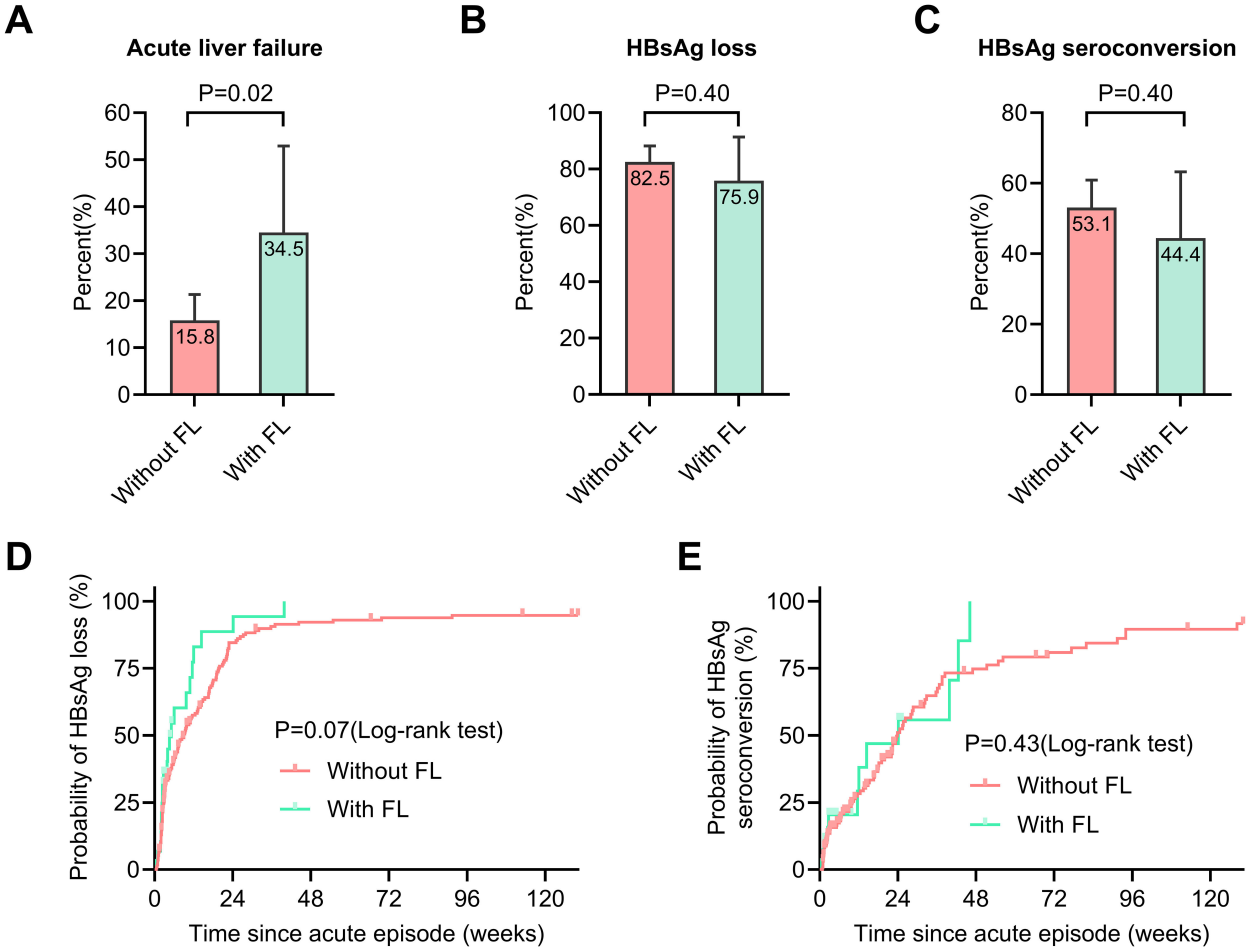
Comparison of primary and secondary outcomes between patients with and without fatty liver. The incidence of acute liver failure **(A)**, HBsAg loss rates **(B)**, and seroconversion rates **(C)** are compared between patients with and without fatty liver. Kaplan-Meier curves illustrate the cumulative incidence of HBsAg loss **(D)** and HBsAg seroconversion **(E)** in both patient groups. The error bars represent the 95% confidence interval estimates of the rates. Abbreviations: FL, fatty liver; HBsAg, hepatitis B surface antigen.

ALF occurred more frequently in AHB-FL patients compared to AHB-no FL patients (34.5% vs. 15.8%, p=0.02) (**Figure 2A**). However, there were no significant differences in HBsAg loss (75.9% vs. 82.5%, p=0.40) and HBsAg seroconversion (44.4% vs. 53.1%, p=0.40) between AHB-FL and AHB-no FL patients (**Figures 2B, C**). The Kaplan–Meier curves further evaluated time to HBsAg loss and seroconversion, and the between-group differences were not statistically significant (log-rank P = 0.07 for HBsAg loss and P = 0.43 for seroconversion) (**Figures 2D, E**). Overall, these results indicate that concomitant FL is associated with a higher risk of ALF in patients with AHB, while no statistically significant between-group differences were observed for HBsAg loss or seroconversion in this cohort.

### 3.3 Association of fatty liver with ALF and HBsAg loss or seroconversion

We further evaluate the association of FL with clinical outcomes using progressively adjusted models (**Table 2**). The primary finding reveals a robust and significant association between FL and ALF. In the unadjusted model (model 1), concomitant FL was associated with a 2.81-fold increased risk of ALF (crude OR: 2.81; 95% CI: 1.18–6.69; p=0.02). This association strengthened in model 2 (adjusted OR [aOR]: 4.82; 95% CI: 1.61–14.43; p=0.01) after adjusting for demographic and lifestyle factors, including age, sex, smoking status, and alcohol consumption. The association persisted in the fully adjusted model 3 (aOR: 4.61; 95% CI: 1.26–16.86; p=0.02), which also incorporated laboratory and clinical covariates such as ALT, AST, HBV DNA, albumin, and SO-to-FMC (**Supplementary Tables S1**, **S2**).

**Table 2:** Association of fatty liver with ALF and HBsAg loss or seroconversion in patients with acute hepatitis B.

Outcomes	Model 1		Model 2		Model 3	
	Odds ratio/hazard ratio (95% CI)	P-value	Odds ratio/hazard ratio (95% CI)	P-value	Odds ratio/hazard ratio (95% CI)	P-value
Primary outcome^*^						
Acute liver failure	2.81 (1.18-6.69)	0.02	4.82 (1.61-14.43)	0.01	4.61 (1.26-16.86)	0.02
Secondary outcome^#^						
HBsAg loss	1.51 (0.96-2.38)	0.07	1.67 (1.00-2.80)	0.049	1.60 (0.92-2.78)	0.10
HBsAg seroconversion	1.28 (0.69-2.35)	0.44	1.37 (0.68-2.77)	0.38	1.69 (0.80-3.57)	0.17

^*^Data are presented as odds ratio (95% CI) and p-value. ^#^Data are presented as hazard ratio (95% CI) and p-value.

Model 1 was unadjusted; Model 2 was adjusted for age (as a continuous variables), sex, smoking status, and alcohol consumption (both as binary variables); Model 3 was further adjusted for the variables in Model 2, along with ALT×ULN, AST×ULN, HBV DNA level, albumin, and SO-to-FMC (all as continuous variables).

ALF, acute liver failure; ALT, alanine aminotransferase; AST, aspartate aminotransferase; CI, confidence interval; DNA, deoxyribonucleic acid; HBsAg, hepatitis B surface antigen; HBV, hepatitis B virus; SO-to-FMC, symptom onset to first medical contact time; ULN, upper limit of normal.

Regarding secondary outcomes, AHB-FL showed no significant association with HBsAg loss (adjusted hazard ratio [aHR]: 1.60; 95% CI: 0.92–2.78; p=0.10) or HBsAg seroconversion (aHR: 1.69; 95% CI: 0.80–3.57; p=0.17). These findings indicated that FL was strongly associated with the risk of ALF events in patients with AHB, which might be an important consideration for clinical management in these patients.

### 3.4 Subgroup analysis

**Table 3** presents a subgroup analysis evaluating the association between concomitant FL and the risk of ALF. Although the estimated effect sizes did not reach statistical significance in some stratifications, a consistent association between the presence of FL and ALF was observed across all subgroups. The interaction effect test showed no significant differences across the pre-specified subgroups (sex: male vs. female; smoking status: current smoker vs. non-smoker; alcohol consumption: drinker vs. non-drinker; marital status: single vs. other; HBV DNA level: <5 log_10_ IU/mL vs. >5 log_10_ IU/mL; HBeAg status: positive vs. negative; and SO-to-FMC time: <5 days vs. >5 days) (p for interaction>0.05). However, the positive association was stronger among individuals from rural areas compared to those from urban areas (p for interaction=0.02). This finding may reveal the potential impact of regional differences on the association between concomitant FL and ALF in patients with AHB.

**Table 3:** Subgroup analysis of the association between fatty liver and acute liver failure.

Subgroup	Odds ratio (95% CI) (with vs. without fatty liver)	P-value	P _for interaction_
Sex			0.72
Male (n=97)	3.38 (1.00-11.37)	0.049	
Female (n=103)	7.70 (1.32-45.08)	0.02	
Current smoker			0.45
No (n=154)	3.96 (1.46-10.76)	0.01	
Yes (n=46)	1.89 (0.15-23.25)	0.62	
Alcohol drinking			0.60
No (n=129)	8.43 (1.96-36.27)	0.004	
Yes (n=71)	2.94 (0.66-13.13)	0.16	
Marital status			0.97
Single (n=69)	4.50 (0.89-22.79)	0.07	
Others (n=131)	2.31 (0.82-6.49)	0.11	
Residential area^*^			0.02
Urban (n=81)	1.44 (0.34-6.06)	0.62	
Rural (n=119)	4.40 (1.44-13.46)	0.01	
HBV DNA			0.10
<5 log_10_ IU/mL (n=155)	2.19 (0.81-5.94)	0.12	
≥5 log_10_ IU/mL (n=45)	6.80 (1.06-43.48)	0.04	
HBeAg			0.67
Negative (n=77)	2.57 (0.76-8.62)	0.13	
Positive (n=123)	3.30 (0.76-14.35)	0.11	
SO-to-FMC			0.51
≤5 days (n=102)	1.77 (0.58-5.40)	0.31	
>5 days (n=96)	6.37 (1.49-27.19)	0.01	

Data are presented as odds ratio (95% CI) and p-value.

^*^The urban-rural classification of the county of residence is based on the National Bureau of Statistics' Urban-Rural Classification Scheme for Counties. Abbreviations: CI, confidence interval; DNA, deoxyribonucleic acid; HBeAg, hepatitis B e antigen; HBV, hepatitis B virus; SO-to-FMC, symptom onset to first medical contact time.

## 4 Discussion

In this study, we demonstrate that FL (vs. no FL) is strongly associated with the risk of ALF events in patients with AHB, highlighting concomitant FL as an independent risk factor for ALF. Moreover, the presence of FL did not influence the rates of HBsAg loss or seroconversion. The observed associations indicate that the impact of FL appears to be more specific to hepatocellular injury rather than antiviral immunity. These findings highlight the need for increased vigilance and targeted management strategies in AHB patients with coexisting fatty liver disease.

The increased risk of ALF associated with FL likely arises from synergistic interactions between HBV-driven inflammation and metabolic dysfunction. First, lipid-laden hepatocytes exhibit mitochondrial oxidative stress and impaired autophagy, rendering them more susceptible to HBV-induced apoptosis (Feldstein et al., 2003; Kakisaka et al., 2012). Second, the chronic low-grade inflammation characteristic of FL, marked by elevated interleukin-6 levels and macrophage activation, may exacerbate HBV-related immune-mediated necrosis (Schuster et al., 2018; Peng et al., 2023). Third, FL-associated metabolic stress can impair hepatic regeneration, thereby limiting recovery from acute injury and accelerating progression to ALF (Borrelli et al., 2018; Michalopoulos and Bhushan, 2021). In this study, AHB-FL patients exhibited higher levels of ALT, AST, and bilirubin compared to AHB-no FL patients, providing additional support for these potential mechanisms.

Our study yields a different conclusion from chronic HBV studies, which reported a higher probability of HBsAg loss and seroconversion in patients with concomitant FL (Mao et al., 2023; Wong et al., 2023). This divergence may reflect differences in innate and adaptive immune responses between individuals with acute and chronic HBV infection (Guidotti et al., 2015; Iannacone and Guidotti, 2022). In addition, previous studies have shown that male gender, older age, negative HBeAg, and genotype C are associated with higher HBsAg seroclearance rates (Yeo et al., 2020). The differences in the distribution of these variables and unmeasured potential confounding factors in this study may have influenced the HBsAg loss and seroconversion rates between AHB-FL and AHB-no FL patients.

Subgroup analysis reveals that the association between concomitant FL and ALF is consistent across various subgroups, including sex, smoking status, alcohol consumption, marital status, HBV DNA levels, HBeAg status, and SO-to-FMC. This consistency indicates that FL may remain a significant risk factor for ALF despite differing background factors. Notably, the positive association between FL and ALF is stronger among individuals from rural areas. This phenomenon may reflect differences in socioeconomic development and healthcare resources between urban and rural regions (Zeng et al., 2021).

Given the global prevalence of FL and the endemicity of HBV in regions such as Asia and Africa (Paik et al., 2022; Park et al., 2023), there is a pressing need for integrated management strategies for patients with AHB. We advocate for routine metabolic profiling to identify high-risk subgroups. Pharmacotherapies targeting fatty liver-related pathways, such as FXR agonists to improve bile acid homeostasis and antioxidants to mitigate oxidative stress, warrant further exploration in clinical trials. Additionally, public health initiatives that promote HBV vaccination and encourage lifestyle modifications could synergistically reduce the burden of ALF in patients with AHB.

This study has several limitations. First, its retrospective design introduces an unavoidable risk of selection bias, and unmeasured confounders may influence the outcomes. Second, the imaging-based diagnosis of fatty liver lacks the sensitivity of histopathological assessment, potentially leading to an underestimation of the severity of steatosis. We acknowledge that non-invasive methods for diagnosing FL may be less accurate than liver biopsy. However, invasive procedures like liver biopsy are neither ethical nor practical for large-scale clinical studies. Third, the relatively small sample size within the fatty liver subgroup (n = 29) limits statistical power and may reduce the stability/precision of multivariable and subgroup estimates (as reflected by wide confidence intervals). Therefore, subgroup and interaction findings should be interpreted cautiously and require confirmation in larger cohorts. Finally, this was a single-center cohort of hospitalized patients and may therefore represent a relatively more severe clinical spectrum of AHB; consequently, the findings may not be fully generalizable to non-hospitalized or community-based AHB populations. Addressing these limitations in future research may enhance our understanding of the interplay between concomitant FL and AHB management.

In conclusion, this study synthesizes a coherent logical chain connecting metabolic dysfunction to adverse AHB outcomes, substantiating the claim that FL is a distinct and potent driver of ALF. By strictly controlling for competing risk factors such as age, alcohol consumption, and viral load in our multivariate models, we have effectively weakened the alternative explanation that observed risks are attributable to general demographic variables or lifestyle factors rather than hepatic steatosis itself. The specific association of FL with liver failure—in the absence of impaired viral clearance—strongly supports a mechanism of “metabolic-viral synergistic toxicity” rather than immune modulation. Consequently, these findings elevate FL from a simple comorbidity to a critical prognostic determinant, mandating a clinical approach where early metabolic screening is integral to preventing ALF in patients with acute hepatitis B.

## Data Availability

The original contributions presented in the study are included in the article/**Supplementary Material**. Further inquiries can be directed to the corresponding authors.
