# Supplementary material for: Association between fatty liver and risk of liver failure in patients with acute hepatitis B: a retrospective cohort study
Source: Front Cell Infect Microbiol. 2026 Jan 27;16:1712115. doi: 10.3389/fcimb.2026.1712115 (PMC12886492; doi:10.3389/fcimb.2026.1712115)
Supplement: Supplementary file 1 [file Table1.docx]

**Supplementary Materials**

**Table S1. Variance inflation factors for candidate variables included in the multivariable analysis.**

| Variables | VIF |
| --- | --- |
| Age | 1.5 |
| Sex | 2.0 |
| Smoking | 1.6 |
| Alcohol | 1.9 |
| Marital status | 1.4 |
| Residential area | 1.1 |
| ALT×ULN | 3.9 |
| AST×ULN | 3.7 |
| Albumin | 1.2 |
| HBV DNA | 1.1 |
| SO-to-FMC | 1.4 |

Collinearity was evaluated using Variance Inflation Factors (VIF), with none of the VIF values exceeding 5, indicating the absence of significant collinearity among the variables.

Abbreviations: ALT, alanine aminotransferase; AST, aspartate aminotransferase; DNA, deoxyribonucleic acid; HBV, hepatitis B virus; VIF, variance inflation factors; SO-to-FMC, symptom onset to first medical contact time; ULN, upper limit of normal.

**Table S2. Selection of candidate variables for multivariable models.**

| **Covariates** | **β** | **Se.** | **Exp (beta)** | **95% CI** | **P-value** |
| --- | --- | --- | --- | --- | --- |
| Age | 0.043 | 0.02 | 1.04 | 1.01-1.08 | 0.01 |
| Sex | 0.673 | 0.38 | 1.96 | 0.94-4.12 | 0.07 |
| Smoking | -1.403 | 0.623 | 0.25 | 0.07-0.84 | 0.03 |
| Alcohol | -0.83 | 0.43 | 0.44 | 0.19-1.02 | 0.06 |
| Marital status | 0.57 | 0.42 | 1.76 | 0.78-4.00 | 0.17 |
| Residential area | -0.14 | 0.37 | 0.87 | 0.42-1.79 | 0.71 |
| ALT×ULN | 0.05 | 0.01 | 1.05 | 1.03-1.07 | <0.001 |
| AST×ULN | 0.04 | 0.01 | 1.04 | 1.02-1.06 | <0.001 |
| Albumin | -0.15 | 0.04 | 0.86 | 0.79-0.93 | <0.001 |
| HBV DNA | -0.05 | 0.11 | 0.95 | 0.77-1.18 | 0.66 |
| SO-to-FMC | -0.16 | 0.06 | 0.85 | 0.75-0.96 | 0.01 |

Abbreviations: ALT, alanine aminotransferase; AST, aspartate aminotransferase; CI, confidence interval; DNA, deoxyribonucleic acid; HBV, hepatitis B virus; SE: standard error; SO-to-FMC, symptom onset to first medical contact time; ULN, upper limit of normal.
